# Iodinated contrast medium: Is there a re(n)al problem? A clinical vignette-based review

**DOI:** 10.1186/s13054-020-03365-9

**Published:** 2020-11-10

**Authors:** Karim Lakhal, Stephan Ehrmann, Vincent Robert-Edan

**Affiliations:** 1 Service d’Anesthésie-Réanimation, Hôpital Laënnec, Centre Hospitalier Universitaire, Boulevard Jacques-Monod, Saint-Herblain, 44093 Nantes, France; 2 Médecine Intensive Réanimation, CIC INSERM 1415, CRICS-TriggerSep Network, CHRU Tours, Tours France and Centre d’étude des Pathologies Respiratoires INSERM U1100, Université de Tours, Tours, France

**Keywords:** Contrast media (MeSH: D003287), Intensive care units (MeSH D007362), Drug-related side effects and adverse reactions (MeSH D064420), Tomography scanners, X-ray computed (MeSH: D015898), Percutaneous coronary interventions (MeSH: D062645), Contrast-induced nephropathy, Post-contrast acute kidney injury

## Abstract

As we were taught, for decades, that iodinated contrast-induced acute kidney injury should be dreaded, considerable efforts were made to find out effective measures in mitigating the renal risk of iodinated contrast media. Imaging procedures were frequently either downgraded (unenhanced imaging) or deferred as clinicians felt that the renal risk pertaining to contrast administration outweighed the benefits of an enhanced imaging. However, could we have missed the point? Among the abundant literature about iodinated contrast-associated acute kidney injury, recent meaningful advances may help sort out facts from false beliefs. Hence, there is increasing evidence that the nephrotoxicity directly attributable to modern iodinated CM has been exaggerated. Failure to demonstrate a clear benefit from most of the tested prophylactic measures might be an indirect consequence. However, the toxic potential of iodinated contrast media is well established experimentally and should not be overlooked completely when making clinical decisions. We herein review these advances in disease and pathophysiologic understanding and the associated clinical crossroads through a typical case vignette in the critical care setting.

## Background

The use of iodinated contrast media (CM) to enhance the imaging visualization of anatomical structures is very frequent. However, in clinical practice, whether the benefits of iodinated CM administration outweigh its potential harms is often unclear [1, 2]. Indeed, acute kidney injury (AKI) that follows intravascular administration of CM—also referred to as contrast-associated AKI (CA-AKI)—is a major concern since it is associated with negative outcomes [3–5]. It occurs in 10–20% of critically ill patients [6], depending on several clinical factors including the patient’s condition but also depending on the definition used [3]. Therefore, clinicians are often puzzled [7]: how to reliably estimate the renal risk of iodinated CM infusion in a patient? How big is the burden of CM renal toxicity? Are there means to attenuate this renal risk? Should the imaging procedure, even urgent, be cancelled or postponed?

Within the impressive myriad of scientific articles on the subject [8], several points have been recently reappraised. We herein review these significant advances through a typical patient-centred case vignette in the critical care setting. We focused on adults because available data in the paediatric population are very limited.

## Main text

A 55-year-old male with a history of type 2 diabetes mellitus complicated with ischemic heart disease and chronic kidney disease presents to the emergency department with abdominal pain and chills. The first clinical examination also reveals fever, skin mottling, hypotension and tachycardia. Serum creatinine concentration is 156 µmol.L^−1^ (1.8 mg.dL^−1^). Arterial lactate concentration is 3.7 mmol.L^−1^. As the attending intensivist, you ask for an abdominal iodinated CM-enhanced computed tomography scanner (CT scan) but the radiologist is worried about the risk of CA-AKI.

### What is the renal risk of CM infusion?

**Fig. 1:**
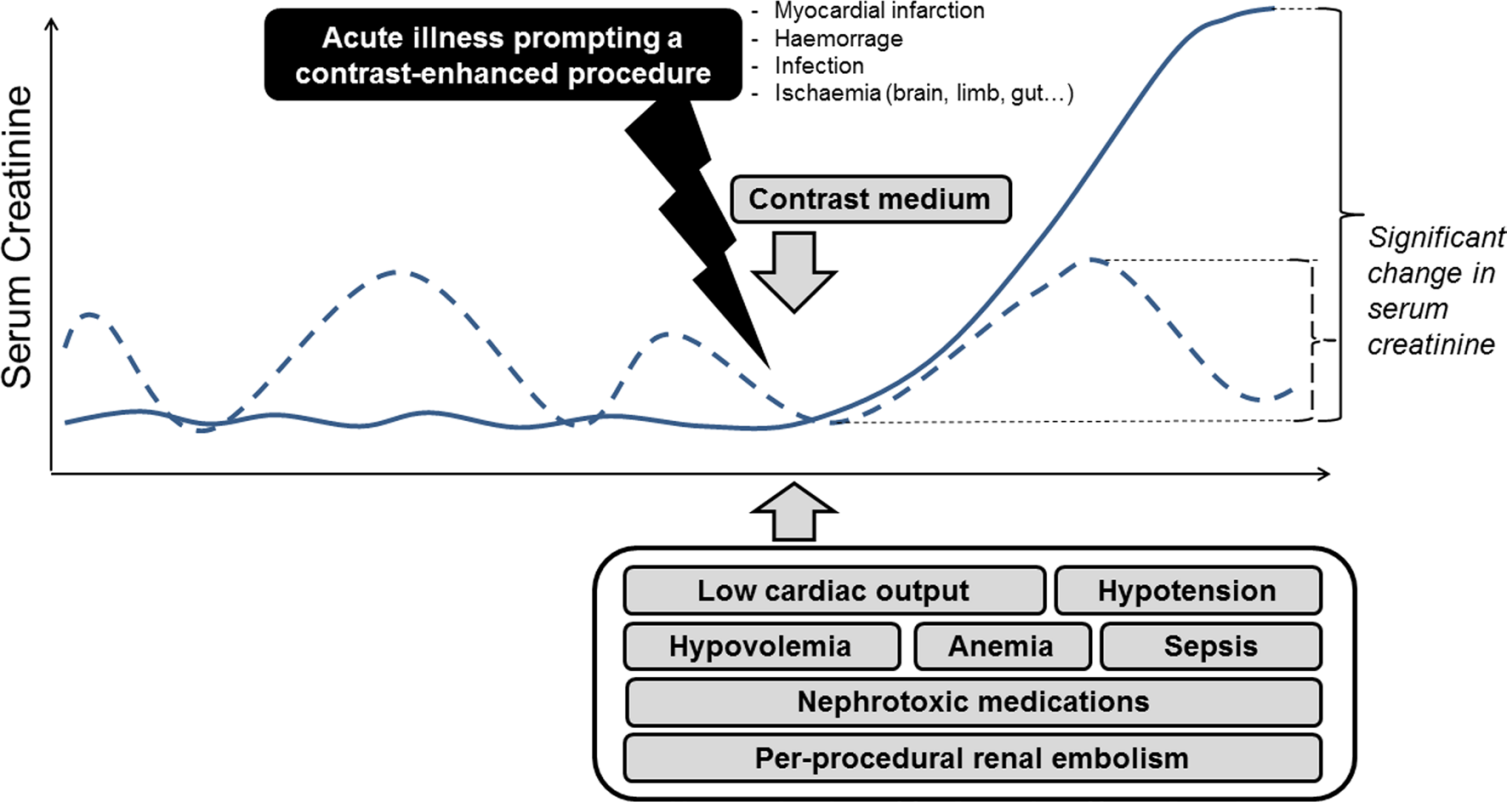
Typical patterns of significant variations of serum creatinine after contrast media infusion with a special emphasis put on alternative causes of acute kidney injury. The acute illness prompting the contrast-enhanced procedure often comes along with several renal insults. Therefore, incriminating the contrast medium in the subsequent decline in renal function is often purely speculative. In addition, the dotted line displays background significant fluctuations of serum creatinine, even before exposure to contrast media. These examples underscore how the determination of contrast medium-specific contribution in the subsequent impairment in renal function is challenging

For decades, fear of contrast-induced AKI has been pervasive and, beyond experimental data, this concern was supported by several clinical studies, mainly in the coronary angiography setting [9]. This belief significantly impacts the decision-making process since some patients may be deprived from a potentially beneficial imaging procedure. Fear of contrast-induced AKI has also prompted tremendous costly efforts to find out means to obviate it [10]. Indeed, hundreds of studies assessed all sorts of preventive measures, most of them being ineffective, some being cumbersome, onerous or even harmful [1]. Importantly, the vast majority of studies assessing the renal risk of CM administration did not include a control group, i.e. patients unexposed to CM but with similar AKI risk factors than exposed patients. Therefore, those uncontrolled studies reported the incidence of AKI following an imaging procedure in critically ill patients rather than the incidence of CM renal toxicity. In our clinical vignette, at the time of the imaging procedure, the patient cumulates several risk factors of AKI: diabetes, chronic kidney disease, potential sepsis, hypotension, low cardiac output, possible other nephrotoxic medications (Fig. 1) [11]. Which one to blame if AKI develops within the few days after exposure to iodinated CM? Is iodinated CM the culprit? May iodinated CM contribute to AKI? To what extent? This uncertainty underscores the importance of a control group in order to determine the respective contribution of iodinated CM and of other risk factors in the development of AKI in the aftermath of the imaging procedure. Hence, among hospitalized patients, meta-analyses of studies including a control group reported that the risk of AKI was similar in patients exposed and unexposed to iodinated CM [12, 13]. This conclusion was consistent in a subgroup of patients particularly at-risk of AKI (patients with diabetes and chronic kidney disease) [12]. Those findings were confirmed among over 6 million patients of a US nationwide registry [14].

One might question the value of these observational controlled studies with direct comparison of patients having received CM with those who have not, invoking a selection bias: CM could have been withheld in patients at risk of AKI. Overcoming this issue is not straightforward. Obviously, an interventional study design that would randomly assign patients to undergo an iodinated CM-enhanced imaging procedure or not would require a very large population [15], may raise ethical concerns and would be source of other biases. Indeed, downgrading the imaging procedure by banning CM administration may worsen patients’ condition and multiple organ failure may arise…including AKI! A comparison of AKI incidence between patients exposed and unexposed to CM would therefore not discriminate the nephrotoxicity of CM from the harmfulness of withholding CM. A more advanced observational study design may be a valuable alternative option: matching patients exposed to CM with unexposed patients having the same baseline risk of receiving CM, using a propensity score approach mimicking randomization to receive CM or not. Observational studies which adopted this design confirmed that the incidence of AKI after the imaging procedure is similar in propensity score-matched exposed and unexposed patients [12, 14]. Likewise, in the specific setting of the intensive care unit (ICU) where AKI is a major concern since renal risk factors are often combined in the same patient, controlled studies using propensity score-matched analysis also failed to demonstrate that iodinated CM precipitates AKI (Table 1). Indeed, retrospective works including thousands of matched patients did not establish causality between CM intravenous exposure and AKI, renal replacement therapy or mortality [16–18]. This was confirmed by a meta-analysis among ICU patients using patient-level data [15] and comprising prospective studies [19, 20]. Similar findings were reported in the emergency department (Table 1).

**Table 1:** Studies comparing the incidence of acute kidney injury between patients exposed and unexposed to intravenous contrast medium in various acute care settings

Reference	Setting/design	Contrast group (patients)	Control group (patients)	Additional means to delineate the renal risk attributable to contrast medium	Comments
Polena et al. [67]	ICU, Retrospective cohort (single-centre)	N = 75 AKI: 18.6%	N = 75 AKI: 2%	None	
Tremblay et al. [68]	Trauma centre; Retrospective cohort (single-centre)	N = 56 AKI: 3%	N = 39 AKI: 16%	None	Proportion of ICU patients unclear
Oleinik et al. [69]	ED, patients with intracerebral haemorrhage; Retrospective cohort (single-centre)	N = 368 AKI: 6% OR 1.4 (95%CI 0.6–3.2)	N = 130 AKI: 14%	Multivariate regression	Proportion of ICU patients unclear
Lima et al. [70]	Stroke patients; Retrospective cohort (single-centre)	N = 575 AKI: 5% OR 0.42 (95%CI 0.24–0.71)	N = 343 AKI: 10%	Multivariate regression	Proportion of ICU patients unclear
Aulicky et al. [71]	Stroke patients; Retrospective cohort (single-centre)	N = 164 AKI: 3%	N = 77 AKI: 4%	Multivariate regression	Proportion of ICU patients unclear
Mc Gillicuddy et al. [72]	Trauma centre, elderly trauma patients; Retrospective cohort (single-centre)	N = 822 AKI: 1.9%	N = 249 AKI: 2.4%	None	Proportion of ICU patients unclear
Ng et al. [73]	ICU patients (oncology); Retrospective cohort (single-centre)	N = 81 AKI: 17%	N = 81 AKI: 17%	1-to-1 matching on baseline serum creatinine, SOFA score and age	
Cely et al. [19]	ICU; Prospective cohort (single-centre)	N = 53 AKI: 9.4%	N = 53 AKI: 15%	1-to-1 matching on baseline creatinine clearance, diabetes, mechanical ventilation, vasopressor use	
Sinert et al. [74]	ED patients with normal renal function; Retrospective cohort (2 centres)	N = 773 AKI: 5.7%	N = 2956 AKI: 9.0%	None	
Kim et al. [75]	ICU, trauma patients; Retrospective cohort (single-centre)	N = 389 AKI: 30% OR 0.99 (CI 95% 0.78–1.25)	N = 182 AKI: 29%	None	
Ehrmann et al. [20]	ICU; Prospective cohort (2 centres)	N = 146 AKI: 5.5%	N = 146 AKI: 5.5%	1-to-1 propensity score matching**	
Christ et al. [76]	ICU patients after cardiac arrest; Retrospective cohort	N = 89 AKI: 15.7%	N = 53 AKI: 37.7%	None	
Gao et al. [77]	ICU; Retrospective cohort (single-centre)	N = 474 AKI: 14.8% OR 1.66 (95% CI 0.72–3.90)	N = 1,896 AKI: 12.4%	Multivariate regression	
Sonhaye et al. [78]	ED; Prospective cohort (single-centre)	N = 620 AKI: 3% OR: 95%CI is missing	N = 672 AKI: 2%	Multivariate regression	Proportion of patients admitted to the ICU unclear
Heller et al. [79]	ED patients admitted to the hospital; Retrospective cohort (single-centre)	N = 6954 AKI: 8.6%	N = 909 AKI: 9.6%	None	8% of patients admitted to the ICU
McDonald et al. [16]	ICU; Retrospective cohort (single-centre)	N = 1223 with eGFR > 45 AKI: 31% OR 1.21 (CI 95% 0.87–1.68) N = 285 with eGFR ≤ 45 AKI: 50% OR 0.88 (CI95% 0.75–1.05)	N = 1223 with eGFR > 45 AKI: 34% N = 285 with eGFR ≤ 45 AKI: 45%	1-to-1 propensity score matching**	An increased risk of dialysis was observed in patients with pre-contrast eGFR ≤ 45 ml/min/1.73 m^2^
Hinson et al. [25]	ED patients admitted to the hospital; Retrospective cohort (single-centre)	N = 7,201 AKI: 6.8% OR 1.00 (95% CI 0.99–1.01)	N = 10,733 AKI: 8.5%	1-to-1 propensity-score matching**	ED critical care designation in 9% of patients Proportion of patients admitted to the ICU unclear
Miyamoto et al. [17]	ICU, patients with sepsis-associated AKI receiving continuous RRT; Retrospective cohort (national database)	N = 3485 Composite outcome (in-hospital death or RRT dependence at discharge): 49.6% OR 0.98 (95% CI 0.88–1.07) RRT dependence at discharge: 4.4% OR 1.08 (95% CI 0.85–1.31) median duration of RRT: 4 [IQR 2–11] days	N = 3485 Composite outcome (in-hospital death or RRT dependence at discharge): 50.2% RRT dependence at discharge: 4.1% median duration of RRT: 4 [IQR 2–11] days	1-to-1 propensity score matching**	
Shih et al. [80]	ICU (subgroup analysis), patients with CKD; Retrospective cohort (national database)	N = 51 30-day RRT: 25.5% aHR 0.95 (95% CI 0.44–2.05)	N = 176 30-day RRT: 25.6%	Cox proportional hazard model adjusted for age, sex and comorbid conditions	Analysis of the Taiwan National Health Insurance Research Database
Goto et al. [81]	ICU (patients with sepsis and AKI); Retrospective cohort (single-centre)	N = 100 further deterioration of renal function = 34%	N = 100 further deterioration of renal function = 35%	1-to-1 propensity score matching**	
Hinson et al. [82]	ED, patients with sepsis; Retrospective cohort (single-centre)	N = 1464 AKI: 7.2 OR 0.99 (95% CI 0.97–1.02)	N = 2707 AKI: 9.6%	1-to-1 propensity score matching**	ED critical care designation in 4% of patients Proportion of patients admitted to the ICU unclear
McGaha et al. [83]	Paediatric trauma centre, severely injured patients; Retrospective cohort (single-centre)	N = 164 AKI: 7.3%	N = 47 AKI: 8.5%	None	57% of patients admitted to the ICU
Williams et al. [18]	ICU; Retrospective cohort (6-hospital health system)	N = 2306 AKI: 19.3% OR 1.11 (95% CI 0.95–1.29)	N = 2306 AKI: 18.0%	1-to-1 propensity score matching**	

*ICU* intensive care unit, *AKI* acute kidney injury*, *Contrast* iodinated contrast media, *OR* odds ratio, *SOFA* sequential organ failure assessment, *95% CI*: 95% confidence interval, *eGFR* estimated glomerular filtration rate, *ED* emergency department, *RRT* renal replacement therapy, *IQR* interquartile range, *CKD* chronic kidney disease, *aHR* adjusted hazard ratio, *DRF* deterioration of kidney function

^*^The definition for AKI may differ from one study to another. This may, in part, account for the between-studies discrepancies in the reported incidence for AKI

^**^Patients exposed and patients unexposed to contrast were matched on their propensity to be administered contrast. This approach aims at mimicking randomization in an observational study design

To assess the specific causal implication of CM among several renal aggressions within hours or days, using a delayed biomarker such as serum creatinine is possibly an imperfect way to proceed. The combination of tissue inhibitor of metalloproteinases-2 (TIMP-2) and insulin-like growth factor binding protein-7 (IGFBP-7) has been proposed as a more specific and sensitive biomarker for early detection of AKI [21]. Interestingly, intravenous CM infusion did not induce significant changes in urinary [TIMP-2]·[IGFBP-7] in ICU patients [22]. This finding may also support the hypothesis of a clinically insignificant toxicity of iodinated CM.

In summary, the renal risk attributable to the intravenous infusion of modern iodinated CM appears, at most, minimal (Table 2). In other words, the widely used term “contrast-induced nephropathy” is often misleading [10]. However, it is unclear whether CM significantly contributes or not to AKI in some very specific populations or during procedures associated with intense kidney exposure to CM (i.e. high systemic dose and/or arterial infusion close to the kidneys). Indeed, patients with pre-existing renal impairment at the time of CM infusion could be more likely to further worsen their renal function if they received iodinated CM [16, 23]. However, this finding of one study, potentially exposed to selection bias [24], has not been firmly confirmed by other studies [25–28] .

**Table 2:** Key messages

Key messages
Nephrotoxicity directly attributable to iodinated CM has been probably exaggerated. One should not refrain from administering CM if deemed necessary
On the other hand, CM is not totally devoid of risks and its use still requires to be wisely weighted
The vast majority of risk factors for CA-AKI are frequent among critically ill patients
Whatever the causal link with iodinated CM, the development of AKI should be anticipated or at least diagnosed early in order to withhold nephrotoxic medications and to adjust the dosage of medications cleared by the kidneys
For the prevention of CA-AKI, as for other causes of AKI, avoiding concomitant renal insults (including withholding nephrotoxic drugs and ensuring correct volemic status) is more effective than initiating specific pharmacological measures which are of no or doubtful utility
Prophylactic RRT seems not justified
There is no need for adapting the schedule of RRT or of the imaging procedure in patients with chronic RRT for end-stage renal disease
Whether intra-arterial administration of modern CM with second-pass exposure is more toxic to the kidney than intravenous CM is unlikely. Uncertainty remains for CM administration with first-pass exposure. Importantly, intra-arterial procedures expose to renal complications which are unrelated to CM toxicity (embolism, circulatory failure, etc.)

*CM* contrast medium, *CA-AKI* contrast-associated acute kidney injury, *AKI* acute kidney injury, *RRT* renal replacement therapy

#### Key message

There is increasing evidence that the nephrotoxicity directly attributable to iodinated CM has been exaggerated. One should therefore not refrain from administering CM if deemed necessary.

Pre-contrast CT-scan shows an enlargement of the right kidney with inflammatory changes and a kidney stone in the urinary tract. These findings are consistent with pyelonephritis with pyonephrosis.

Should the intensivist insist on getting a CM-enhanced CT-scan?

### Experimental data about the renal toxicity of iodinated CM

Several aforementioned controlled studies questioned the clinical relevance of the impact of CM administration on the kidney. However, the in vitro renal toxicity of iodinated CM could hardly be denied, even if many animal models may have poor applicability to humans. CM may be toxic to the kidney through [29]:

- renal ischemia via CM-induced arterial vasoconstriction impairing renal blood flow.
- The release of reactive oxygen species.
- Direct tubular toxicity (osmotic nephrosis, induction of apoptosis, cellular energy failure).

Besides the administered volume, osmolarity of the iodinated CM is of importance. High-osmolar CM, involved in the first historic CA-AKI descriptions, are more nephrotoxic than modern iso- and low-osmolar CM and were therefore abandoned [30].

The aforementioned observational controlled studies reporting a similar incidence of AKI in patients exposed and unexposed to CM suggest that modern CM are of modest clinical impact on renal function. Of note, an alternative interpretation of these studies could be that fear of contrast-induced nephropathy may have prompted a thorough periprocedural management aiming at mitigating the CM-related renal risk: nephroprotective measures, rationale administration of CM (type, volume, timing of administration). Therefore, even if concerns about CM nephrotoxicity will lessen, caution about the renal risk of CM as well as its non-renal risks (e.g. anaphylaxis, additional radiation exposure) will always remain warranted.

In our clinical vignette of acute pyelonephritis due to urolithiasis, the indication for CM administration could be questioned: unenhanced CT can detect calculi, obstruction, renal enlargement, gas formation and inflammatory masses [31]. It was sufficient for the diagnosis of pyelonephritis with pyonephrosis. Since CM-enhanced CT may provide more subtle information [31, 32] but possibly not crucial in the urgent decision-making, the diagnostic yield of CM administration and its therapeutic consequences should be discussed with the radiologist.

#### Key message

Modern CM are less nephrotoxic than older, high-osmolar, iodinated CM. However, considering the renal toxicity of iodinated CM in animal models, besides other related risks (anaphylaxis, additional radiation exposure, etc.), CM cannot be considered totally devoid of risks and should therefore be administered only if necessary.

The radiologist asks you about the patient’s risk factors for CA-AKI.

### Risk factors for CA-AKI

The use of high-osmolar CM, ionic CM or high-viscosity CM is nowadays seldom. The infused volume of CM should be limited to as little as possible [33–35]. The renal impact of repeated exposure to CM within a short period of time should be better evaluated [36].

Patient-related risk factors for CA-AKI are nonspecific, common to other causes of AKI: pre-existing impaired renal function which may be unknown and overlooked, diabetes mellitus, chronic hypertension, advanced age, malignancy, metabolic disorders, anaemia, heart failure, hypovolemia, hypotension, inflammation/sepsis [33–35]. Special emphasis should be put on concurrent nephrotoxic medications [11, 20, 37]. For instance, patients treated with angiotensin-converting enzyme inhibitors or angiotensin receptor blockers are more prone to develop CA-AKI [38]. Anyhow, these drugs are usually withheld when an acute severe illness occurs, irrespective of CM administration, but residual effect may last at the time of the imaging procedure. Periprocedural administration of diuretics has been advocated by some authors as a means to mitigate the renal toxicity of CM (for instance, via the reduction of exposure to CM by increasing urine flow) [39]. However, diuretics promote negative fluid balance and thereby CA-AKI if fluid losses are not promptly replaced [39]. The prescription of some nephrotoxic medications can be hardly avoided as it can be dictated by the acute condition which prompted the imaging procedure such as anti-infective agents, for instance (aminoglycoside, vancomycin, high-dose beta-lactams, amphotericin B, acyclovir, etc.) [11].

The patient is currently treated with metformin. Metformin is the typical example of a drug that may accumulate if a decline in renal function occurs. Dosing adjustment of drugs cleared by the kidney is therefore desirable. To prevent metformin-associated lactic acidosis, although this complication has probably been overestimated [40], metformin should be withheld if a decline in renal function is likely to occur or has occurred [41]. In our clinical vignette, irrespective of CM administration, sepsis-induced AKI was a sufficient reason to withhold metformin.

Unsurprisingly for a critically ill patient, several risk factors for AKI are present in our clinical vignette. Pragmatically, one may consider all critically ill patients at risk of AKI. In addition, common sense dictates that, when a patient is admitted to the hospital with high serum creatinine, looking for a pre-existing chronic kidney disease (by enquiring about serum creatinine measurements within the previous weeks/months) is advisable in order to estimate baseline renal function as well as investigating differential diagnosis of possible chronic kidney disease.

#### Key message

The vast majority of the risk factors for CA-AKI are frequent in the critically ill. Whatever the causal link with iodinated CM, the development of AKI should be anticipated or at least diagnosed early in order to withhold non-indispensable nephrotoxic medications and to adjust the dosage of medications cleared by the kidneys.

Whatever, the patient received intravenous CM. A medical student suggests acetylcysteine administration to prevent AKI and ask you about other preventive measures.

### CA-AKI pharmacological prophylaxis

Almost 200 randomized controlled trials (RCTs) tested pharmacological prevention of CA-AKI [42]. More than 42,000 patients were enrolled in these RCTs. N-acetylcysteine (> 6000 patients), intravenous hydration (> 5000), sodium bicarbonate (≈ 3400), statins (> 3000) were the most tested among more than 40 tested interventions [42]. For each prevention method, conflicting conclusions were reported, from one study to another and even from one meta-analysis to another [43–45]. Recently, a large RCT (≈ 5000 patients at risk of CA-AKI, i.e. with pre-existing impaired renal function) has shown neither a superiority of N-acetylcysteine over placebo nor a superiority of IV sodium bicarbonate 1.26% over IV saline 0.9% for a scheduled angiography (coronary or other) [46]. In ICU patients, similar results were reported for N-acetylcysteine [47] and sodium bicarbonate [48] and are therefore not recommended [24]. Of note, in ICU patients with both moderate-to-severe AKI of various causes and severe metabolic acidaemia (pH ≤ 7.20), therapeutic-rather than prophylactic-administration of sodium bicarbonate was associated with a higher incidence of survival by day 28 [49].

Maintaining a correct hydration status is a mainstay of the prevention of AKI [24]. However, it is worth reminding that excessive hydration could be harmful. Indeed, in non-ICU patients (> 90% of outpatients) with impaired renal function (estimated glomerular filtration rate of < 60 mL/min/1.73 m^2^) and undergoing an elective procedure with CM administration, prophylactic administration of intravenous 0.9% NaCl did not reduce the incidence of CA-AKI and 4% of patients developed signs of congestive heart failure and hydration had to be stopped prematurely, diuretic therapy had to be prompted, and/or hospitalization has been extended [50].

None of the other pharmacological prevention methods was associated with robust evidence of benefit whereas their possible harmfulness should be kept in mind.

Failure to undoubtedly demonstrate positive effects of different prophylactic strategies targeting various pathways of CM renal possible toxicity could be related to the little clinical impact of this toxicity itself with modern CM. It is therefore likely that most studies testing these strategies were underpowered or that the assumptions made about the tested preventive measures were erroneous.

#### Key message

For the prevention of CA-AKI, as for other causes of AKI, avoiding concomitant renal insults (including withholding unnecessary nephrotoxic drugs and ensuring correct volemic status) is more effective than initiating specific pharmacological measures which are of no or doubtful utility.

Before the admission to the ICU, antimicrobial therapy was initiated, along with fluid resuscitation and norepinephrine. A ureteral stent has been inserted. The fellow proposes to start renal replacement therapy to remove CM.

### Renal replacement therapy after contrast administration

Besides considering RRT a few days after CM infusion, if severe AKI developed, some authors investigated RRT for the *prevention* of CA-AKI, i.e. to clear CM from the patient’s blood. Hence, prophylactic RRT has been tested in the immediate aftermath of CM infusion or even during the CM-enhanced imaging procedure [51]. Indeed, RRT increases the clearance of iodinated CM [51, 52]. Accelerating the withdrawal of CM from the body may be appealing in patients with severe renal dysfunction: the lower the glomerular filtration rate the higher the half-life of iodinated CM, yielding a high concentration of CM during a protracted period [52]. Iodinated CM are water-soluble and their extracellular distribution, their limited protein binding and their lack of metabolization could make RRT a suitable means to remove CM from the body [52]. However, despite an effective CM removal, RRT does not reduce the incidence of CA-AKI according to a meta-analysis of 11 single-centres studies among 1010 patients with mild to severe chronic kidney disease (CKD), predominantly undergoing coronary angiography [51]. RRT modality and baseline renal function may matter since prophylactic haemodialysis was even associated with a higher risk of CA-AKI in patients with mild CKD as compared with standard medical therapy whereas it was not the case in the scarce studies addressing prophylactic haemofiltration or prophylactic haemodialysis in patients with more severe CKD [51, 53]. Overall, possible explanations for the lack of clear nephroprotective effects of prophylactic RRT are RRT-specific risks at the renal level (blood-membrane contact-related inflammation, systemic hypotension), an insufficient removal of CM before the onset of renal injury [54] and the lack of clinically significant toxicity of CM.

#### Key message

Prophylactic RRT should not be performed since this overuse of medical resources seems not justified [41].

Another common issue is “when to perform RRT in patients undergoing chronic RRT?”. Indeed, scheduling a RRT session immediately after the exposition to CM is a frequent request to nephrologists or intensivists, in an attempt to prevent further renal damage or even extra-renal complications of the delayed excretion of CM. However, there is no evidence of the benefit of this resource mobilizing strategy [41, 55].

#### Key Message

Unless the periprocedural management of the imaging procedure was associated with life-threatening fluid overload (not fully related to the CM itself), there is no need for adapting the schedule of RRT or of the imaging procedure in patients with chronic RRT for end-stage renal disease.

Two days later, sepsis is under control, but the patient complains of chest pain. The electrocardiogram reveals an ST segment elevation and serum cardiac troponin has dramatically increased, suggesting acute myocardial infarction (STEMI). A percutaneous coronary intervention (PCI) is considered. Does the renal risk differ between iodinated CM administration for STEMI and for CT scan?

### Intra-arterial versus intravenous CM and renal risk

Intra-arterial administration of CM is often deemed to be associated with a higher renal toxicity than the intravenous route [35], but this belief is somewhat controversial [56]. When comparing intravenous to intra-arterial CM infusion in the same patient (the patient being his own control), the route of administration does not seem to impact the incidence of post-procedural AKI [57–60], even if these retrospective studies were possibly exposed to bias [33]. Determining the toxicity of CM with respect to the route of administration cannot be straightforward. Indeed, beyond this route, the incidence of post-procedural AKI greatly depends on the patient’s condition [33]. Indeed, an elective PCI is associated with a markedly lower risk for AKI (1–2%) than a PCI for STEMI (incidence of 10–20%) [1]. To complicate matters, AKI following intra-arterial procedures may be related to catheter-related insults to the kidney rather than CM toxicity. For instance, in more than 50% of PCIs, the placement of guiding catheters is source of scraping debris from the aorta, exposing the kidney to atheromatous emboli-related ischemia [29]. In addition, impaired renal perfusion is not rare in this setting, due to fluid restriction, arrhythmias, myocardial infarction. These events may lead to a decline in renal function that may be erroneously ascribed to the CM itself, not to mention, again, the concurrent conditions and treatments that also affect renal function [61, 62]. Hence, as for the intravenous route, the clinical relevance of CM-induced nephropathy following an intra-arterial administration is controversial. Interestingly, in the specific setting of PCI which is the most studied intra-arterial CM administration, observational controlled studies failed at firmly demonstrating that iodinated CM precipitates AKI. Patients with STEMI exposed to iodinated CM (during PCI) had a similar incidence of AKI than propensity-matched control patients (treated with thrombolysis or without reperfusion) [63]. In patients with non-ST-elevation acute coronary syndrome, one study reported that performing PCI within 2 days of hospital admission was associated with a small increase in the risk of AKI but not in the risk of dialysis or long-term progression to end-stage renal disease [4]. In another study among patients with high renal risk, cardiac angiography was followed by a very low incidence of CA-AKI episodes associated with serious adverse outcomes over a 3-month follow-up [64].

As illustrated by a study unexpectedly reporting a lower rate of AKI in patients exposed to CM during PCI than in unexposed patients [14], it is likely that such observational propensity-matched controlled studies tested the renal impact of early invasive management of acute coronary syndrome rather than the renal toxicity of iodinated CM. This illustrates how determining the specific role of CM in CA-AKI is difficult. However, this finding underscores that depriving the patient from an imaging procedure may be more harmful than exposing him to the potential renal toxicity of CM. Again, the renal toxicity of iodinated CM—or at least of some iodinated CM—is undeniable, as suggested by some meta-analyses of randomized controlled trials comparing low- and an iso-osmolar CM (iodixanol) in the subgroup of coronary procedures indicating that CA-AKI was more frequent with low-osmolar CM [65, 66]. This was not the case with intravenous administration [65]. Overall, one may consider that, with modern CM, even used intra-arterially, the burden of CA-AKI has been exaggerated [1].

Last, within intra-arterial CM administrations, it is important to distinguish *second-pass* exposure, in which the CM reaches the kidneys in a diluted form, from *first-pass* exposure in which the kidney is exposed to a relatively undiluted CM. The former occurs when CM is infused in the right heart, the pulmonary arteries, selectively in branches of the suprarenal aorta (carotid, subclavian, brachial, coronary and mesenteric arteries) or in the infrarenal aorta and its branches. The latter occurs when CM is infused into the left heart, the suprarenal aorta and, selectively, into the renal arteries. In recent guidelines, intra-arterial CM administration with second-pass exposure was considered to have no higher renal toxicity than intravenous CM administration [33]. As aforementioned, there is no strong evidence that, during coronary angiography and PCI which frequently combine both first- and second-pass renal exposure, the toxicity of CM is higher. However, as a precaution and because of catheterism-related complications, a more thorough prevention of AKI is recommended [33].

#### Key Message

Whether intra-arterial administration of modern CM with second-pass exposure is more toxic to the kidney than intravenous CM is unlikely. Uncertainty remains for CM administration with first-pass exposure. Importantly, intra-arterial procedures expose to renal complications which are unrelated to CM toxicity (embolism, circulatory failure, etc.). Intra-arterial procedures can convey important patients benefit per se.

## Conclusions

Despite thousands of papers addressing CA-AKI, several areas of uncertainty persist. However, recent advances tended to shake up some old beliefs about CA-AKI. Indeed, the actual burden of CM toxicity has been exaggerated for years, mostly owing to the coexistence of other renal insults at the time of CM administration (nephrotoxic medications, sepsis, abnormal circulatory status, renal embolism during intra-arterial procedures, etc.) which predominantly contributed to the decline in renal function in the aftermath of CM administration. As a consequence of such overrating, beyond flawed epidemiologic data, several studies assessing pharmacological measures to prevent CA-AKI were probably underpowered. Rather than administering drugs of doubtful benefit, a rational use of modern CM and avoidance/limitation of concomitant renal insults should be the mainstays of CA-AKI prevention and treatment.

## Data Availability

Not applicable.

## Abbreviations

AKI: Acute kidney injury
CA-AKI: Contrast-associated acute kidney injury
CKD: Chronic kidney disease
CM: Contrast medium
CT scan: Computed tomography scanner
ICU: Intensive care unit
PCI: Percutaneous coronary intervention
RCT: Randomized controlled trial
RRT: Renal replacement therapy
[TIMP-2]·[IGFBP-7]: Combination of tissue inhibitor of metalloproteinases-2 and insulin-like growth factor binding protein-7
STEMI: ST-elevation myocardial infarction
